# Personality Traits and E-learning Course Satisfaction: A Study of Health Science Students

**DOI:** 10.7759/cureus.89131

**Published:** 2025-07-31

**Authors:** Asieh Ashouri, Mahdokht Taheri, Mahboobeh Rasouli, Sepideh Rouhalamin

**Affiliations:** 1 Educational Development Center, Medical Education Research Center, Guilan University of Medical Sciences, Rasht, IRN; 2 Health and Environment Research Center, School of Health, Guilan University of Medical Sciences, Rasht, IRN; 3 Department of Biostatistics, School of Public Health, Iran University of Medical Sciences, Tehran, IRN

**Keywords:** big five inventory, e-learning, online course satisfaction survey, personality traits, satisfaction, students

## Abstract

Background

Personality traits influence various aspects of life, including educational experiences, and learner satisfaction is a crucial determinant of an effective educational system. Understanding the relationship between learners’ personality traits and their satisfaction with e-learning courses may highlight the need for tailored instructional designs that accommodate individual differences and enhance the personalized educational experience. This study aimed to investigate how different personality traits, based on the Big Five Personality Inventory (BFI), correlate with students’ satisfaction in e-learning environments.

Methodology

In this cross-sectional study conducted in 2022, data were collected from health faculty students who had utilized e-learning for a minimum of one academic semester. Participants self-reported their demographic information, completed the BFI (44-item version) questionnaire, and filled out the Online Course Satisfaction Survey, including subscales of satisfaction with the (a) instructor, (b) technology, (c) course setting, (d) interaction, (e) outcomes, and (f) overall satisfaction. Both instruments are valid and reliable measures. The data were analyzed using hierarchical regression models.

Results

The study involved 183 participants, 138 (75%) of whom were female, with an average age of 23 years. Among the Big Five personality traits, the highest mean scores were observed for agreeableness, followed by openness to experience, conscientiousness, extraversion, and neuroticism. Regarding e-learning satisfaction, the highest to lowest mean scores were recorded for the subscales of technology, course setting, interaction, outcome, instructor, and overall satisfaction. Overall satisfaction was moderate across the different dimensions assessed. Notably, with a small effect size, neuroticism inversely correlated with satisfaction related to the instructor, technology, course setting, interaction, and outcome, while higher agreeableness was associated positively with technology satisfaction (p < 0.05). The regression analysis explained 6% to 28% of the variance in satisfaction depending on the subscale.

Conclusions

Despite small effect sizes, the study identified meaningful associations between personality traits, particularly neuroticism and agreeableness, and e-learning satisfaction. Incorporating psychologically informed strategies into course design may help address diverse learner needs and foster more supportive and effective online learning environments.

## Introduction

In an educational system, learners differ cognitively, psychologically, emotionally, skillfully, behaviorally, and generally in terms of personality. These differences explain how individuals perceive, judge, and behave in certain situations. Costa and McCrae proposed five major personality factors, namely, extraversion, neuroticism, openness to experience, conscientiousness, and agreeableness, based on the personality traits approach [1-3]. Each of these traits influences aspects of individuals’ social, emotional, and cognitive behavior, and although they may vary to some extent across the lifespan, they are generally considered relatively stable [3].

Studies indicate that individuals’ personalities can affect their academic success, careers, social relations, physical health, and well-being [3]. For example, Ghorbani et al. [4] reported that learners’ introversion is negatively correlated with the overall grade point average (GPA), whereas emotionally unstable extraverted individuals have significantly greater academic achievement.

In computer-based learning, learners’ personalities play a significant role in their study behaviors, communication with instructors and peers, and learning preferences [5]. Some studies suggest that virtual learning environments may be more compatible with introverted students’ behaviors, facilitating greater participation and social presence, which could lead to their greater success [2,3,6]. Momeni Rad et al. [6] reported that introverted students participated significantly more than extroverted students in Moodle learning management systems.

However, research findings on the relationship between personality traits and academic performance have been inconsistent. For example, Katrimpouza et al. [7] found associations between conscientiousness and openness to experience with academic social media activity, but reported no significant link to academic performance. In contrast, a doctoral thesis conducted in Iran revealed that neuroticism was negatively correlated with academic achievement among dental students, whereas conscientiousness was positively correlated. However, no such relationship was observed among medical students (Doctoral thesis: Khanzadeh H. Comparison of Personality Traits of Senior Students of Medicine and Dentistry and Its Relation With Academic Achievement in Guilan University of Medical Sciences; 2021).

Although the ultimate goal of education is learning, learner satisfaction is also a key indicator of an educational system’s success. Satisfaction is defined as the extent to which students perceive their learning experience as fulfilling their needs and expectations. In e-learning environments, satisfaction is described as “the degree of satisfaction students have with the entire e-learning system” [8]. Learner satisfaction can lead to higher levels of motivation, engagement, learning, performance, and success [9,10].

Studies have shown that personality traits may influence students’ satisfaction with virtual learning environments. Bolliger and Erichsen [11] reported that personality types significantly influenced student satisfaction in both online and blended learning settings. Similarly, research employing data mining techniques has emphasized the role of individual differences, such as personality traits, in shaping the quality of e-learning experiences [12].

A theoretical framework guiding this study integrates the Five-Factor Model of personality with the Technology Acceptance Model. The Five-Factor Model, which describes how personality traits influence individual behavior, serves as the foundation for understanding how learners’ characteristics shape their interactions with e-learning environments. Specifically, personality traits are hypothesized to influence students’ learning preferences, coping strategies, and communication styles within online settings [10,13]. On the other hand, the Technology Acceptance Model emphasizes how students’ perceptions of the usefulness and ease of use of the e-learning system impact their engagement and satisfaction [8,14]. By integrating these two models, the study suggests that personality traits may indirectly influence learners’ perceptions of technology, which, in turn, affects their engagement, motivation, overall learning experience, and satisfaction with e-learning. Thus, we propose that learners’ satisfaction is not only determined by the functionality of the e-learning platform but also by how their intrinsic personality traits shape their approach to, and interaction with, digital environments and e-learning courses.

Research on e-learning satisfaction has focused primarily on factors such as course design, instructional quality, and technological usability [5,15,16]. Furthermore, various studies have examined learner-related factors, including age, gender, prior experience with technology, and learning styles, as influencing factors of satisfaction levels [15]. However, the influence of personality traits on satisfaction within e-learning settings has received limited attention [15,16].

Since the use of e-learning has become widespread during and after the COVID-19 pandemic, students with a variety of personality traits may have engaged in e-learning or other technology-based educational methods. Consequently, conducting studies to optimize the delivery of these types of instruction has become increasingly necessary. Currently, few studies have investigated the relationships between personality traits and satisfaction with electronic educational environments, which often yield contradictory results [17]. Furthermore, most studies have been conducted on students outside of the health sciences, resulting in a lack of research specifically targeting this learner population, who may face specific challenges in their online learning experiences [14].

Therefore, this study was conducted to investigate the relationships between personality traits and e-learning course satisfaction among health science students. By providing empirical evidence on the impact of personality traits on satisfaction derived from the design and delivery of e-learning courses, this research contributes to the existing body of literature. Acquiring knowledge in this field can provide insights for future research aimed at personalizing and optimizing electronic teaching and learning processes, as well as guiding e-learning educators in developing targeted interventions to increase learner satisfaction and success.

## Materials and methods

Study design and ethical approval

This study was conducted in a cross-sectional manner in the health faculty of Guilan University of Medical Sciences (GUMS). The study was approved by the GUMS Research Vice-Chancellor and the Ethics Committee (approval number: IR.GUMS.REC.1401.368). Before participating in the study, students were informed about the purpose of the study and their right to voluntarily participate and withdraw at any time without penalty. Informed consent was obtained from all students who agreed to participate in the study.

Participants

In a consensus manner, all continuous or discontinuous bachelor’s degree students who had undergone e-learning for at least one academic semester (using the Navid e-learning management system and participating in online classes) and were willing to participate in the study were included. The students received e-learning throughout the academic years from 2019 to 2022 during or after the outbreak of COVID-19. Despite conducting the study as a census, the minimum sample size required to evaluate the study hypotheses was calculated. The minimum sample size was estimated to be 176 individuals, based on a correlation of 0.30 [6,18], a confidence level of 95%, a power of 90%, and a 20% compensation for missing data, as well as an increase of 40 samples for examining the five different personality traits in the regression analysis.

Instruments

Data on learners’ demographic and educational characteristics, personality traits, and satisfaction with the e-learning courses were recorded via an electronic questionnaire. Demographic characteristics included age, sex, marital status, major, and place of residence. Educational characteristics included degree, semester, GPA, tools used to access the course, and perceived adequacy of hardware resources and software skills.

To measure personality traits, the short version of the Big Five Inventory (BFI-44), developed by John and Srivastava, was used. This questionnaire consists of 44 items rated on a five-point Likert scale ranging from completely disagree (1) to completely agree (5), and includes the following five personality factors: extraversion, agreeableness, conscientiousness, neuroticism, and openness to experience. The sum of the scores for each personality trait was calculated. The validity and reliability of the Persian version of this questionnaire were evaluated and confirmed in a study by Nosratabadi et al. [19].

Satisfaction with the e-learning course was measured via the Online Course Satisfaction Survey (OCSS) questionnaire developed by Bollinger and Halupa [20]. The questionnaire contains items rated on a five-point Likert scale ranging from 1 (strongly disagree) to 5 (strongly agree) across the subscales of satisfaction with the (a) instructor, (b) technology, (c) course setting, (d) interaction, (e) outcomes, and (f) overall satisfaction. Higher summation values on each subscale indicate greater satisfaction. First, the questionnaire was translated into Persian, and its content validity was evaluated by a panel of experts, which included seven faculty members specializing in medical science education or e-learning. Two items related to satisfaction with the use of discussions and/or forums and satisfaction with the use of chat tools were merged based on contextual considerations. Finally, the questionnaire, which includes 28 items with an average content validity ratio of 0.87 and a content validity index of 0.91, was approved. The reliability of the questionnaire was also confirmed by a sample of 13 students, yielding a Cronbach’s alpha coefficient of 0.72 for satisfaction with the instructor subscale, 0.62 for satisfaction with technology, 0.79 for satisfaction with the course setting, 0.86 for satisfaction with interaction, 0.71 for satisfaction with outcome, and 0.76 for satisfaction with overall e-learning. In all study participants (N = 183), the Cronbach’s alpha coefficient was 0.84, 0.72, 0.79, 0.82, 0.76, and 0.87 for these subscales, respectively.

Data collection

After providing informed consent, data were gathered via a self-administered electronic questionnaire sent to each student of the faculty. The link to the electronic questionnaire was sent to each class group of students via a social networking application (WhatsApp) through the class representative, as well as directly to each student via SMS. In addition to providing demographic and educational information, students were asked to express their satisfaction with the e-learning courses they had experienced, either currently or in previous academic semesters, by completing the OCSS questionnaire. They were also requested to fill out the BFI-44 questionnaire based on their actual traits and characteristics, as they are, rather than how they would like to be. The reminders to complete the questionnaire were sent once each week, twice over two weeks. Data collection was conducted in December 2022.

Statistical analysis

The data were analyzed via SPSS Software version 26 (IBM Corp., Armonk, NY, USA). The frequency (percentage), mean (standard deviation (SD)), or median (minimum and maximum) were used to describe qualitative or quantitative data. The normality of the quantitative data was checked via the Shapiro-Wilk test and Q-Q plot, and the results were confirmed for all variables except for student age. To further describe personality traits, the values were grouped into the following three levels based on the terciles of the possible range of 1 to 5: low (1 to 2.33), moderate (2.33 to 3.66), and high (3.66 to 5). This grouping was used solely for descriptive purposes.

To examine the relationships between students’ demographic and educational variables and their satisfaction scores with e-learning courses, multivariate analyses of variance were conducted. In these analyses, the continuous variables of age and semester were categorized. Hierarchical multiple regression models were used to investigate the relationships between personality traits and satisfaction. Demographic characteristics were entered into the model in a stepwise manner, and only significant characteristics were included in the model at the first level. Personality trait variables were included in the model at the second level. This approach assessed the incremental contribution of personality traits to satisfaction after controlling for participants’ demographic variables. Continuous variables such as personality traits, age, and semester were entered into the regression models without being categorized. The assumptions of a normal distribution of residuals, homogeneity of variances, non-collinearity between variables, and non-autocorrelation of residuals were checked and satisfied in all regression analyses. The significance level was set at 0.05.

## Results

Participant demographics

Among the 341 eligible students from the GUMS health faculty, 183 (54%) participated in the study. Given the response rate of 54%, the demographic distribution of the participants was consistent with that of the overall student population in the health sciences faculty at GUMS, suggesting that the sample was representative. Descriptions of the students’ characteristics are provided in Table 1. Among all the participants in the study, 138 (75%) were female and 45 (25%) were male. The median age was 23 years, ranging from 18 to 53 years. The majority of the students (n = 137, 75%) were single, and fewer than half of the students (n = 73, 40%) lived in the dormitory. The average GPA reported by the students was 16.9 (SD = 1.36; range = 12.3-19.8). The students stated that they most commonly used a smartphone (n = 103, 56%), followed by a computer or laptop (n = 83, 45%), and printed handouts or hardcopy books (n = 74, 40%) to read or learn their course content during e-learning. In total, 34 (19%) students perceived their access to hardware facilities as insufficient, and 17 (9%) students perceived their software skills as inadequate for effective participation in e-learning.

**Table 1:** Characteristics of the study population (n = 183). Data are frequency (percent), otherwise indicated. The difference from the total of 183 is due to missing data. *: GPA was recorded based on the students’ statements. **: Up to two options could be selected, and percentages in each row were calculated based on the total number of participants (N = 183).

Characteristic	Number of students (%)
Gender	
Female	138 (75)
Male	45 (25)
Age in years	
18–21	63 (34)
22–24	64 (35)
>24	56 (31)
Marital status	
Single	137 (75)
Married	46 (25)
Major	
Public Health	81 (49)
Occupational Health	31 (19)
Environmental Health	53 (32)
Education level	
Continuous BSs	129 (70)
Non-continuous BSc	54 (30)
Semester	
1–2	28 (15)
3–4	73 (40)
5–6	45 (25)
7–8	37 (20)
Living status	
With family	59 (32)
Private	51 (28)
Dormitory	73 (40)
Total GPA*	
<16	49 (28)
16–17.5	78 (45)
>17.5	48 (27)
Most often used media in learning**	
Computer/Notebook/Laptop	83 (45)
Hardcopy/Printed materials or books	74 (40)
Smartphone	103 (56)
Free sources on the internet	6 (3)
Perceived access to hardware facilities	
Not enough	34 (19)
Relatively enough	97 (53)
Totally enough	52 (28)
Perceived software skills ability	
Not enough	17 (9)
Relatively enough	102 (56)
Totally enough	64 (35)

Students’ personality traits

Descriptions of the students’ personality traits are provided in Table 2. The personality traits of agreeableness, openness to experience, conscientiousness, extraversion, and neuroticism had mean scores ranging from the highest to the lowest.

**Table 2:** Description of the Big Five personality traits of the study population (n = 183).

Personality trait	Median	Mean	SD	Minimum	Maximum	Low trait score, n (%)	Moderate trait score, n (%)	High trait score, n (%)
Openness to experience	3.60	3.56	0.54	2.10	4.80	3 (2)	103 (56)	77 (42)
Neuroticism	2.75	2.79	0.72	1.00	4.88	53 (29)	105 (57)	25 (14)
Conscientiousness	3.44	3.49	0.52	2.22	4.89	1 (1)	108 (59)	74 (40)
Agreeableness	3.78	3.69	0.52	2.00	4.89	2 (1)	74 (41)	107 (58)
Extraversion	3.12	3.10	0.60	1.50	4.50	21 (11)	130 (72)	32 (17)

E-learning course satisfaction

Satisfaction with the e-learning course across all dimensions was moderate, with mean scores ranging from 2.80 to 3.36. Satisfaction with technology, course setting, interaction, outcome, instructor, and overall scores ranged from the highest to the lowest (Table 3).

**Table 3:** Description of students’ satisfaction with the e-learning course (n = 183).

Satisfaction	Median	Mean	SD	Minimum	Maximum
Instructor	3.17	3.10	0.88	1	5
Technology	3.50	3.36	0.69	1.25	5
Set-up	3.25	3.26	0.77	1.50	5
Interaction	3.17	3.17	0.82	1	5
Outcomes	3.25	3.13	0.95	1	5
Overall	3.00	2.80	1.06	1	5

Relationships between demographic and educational characteristics and satisfaction with e-learning courses

Assessing the relationships between students’ demographic and educational characteristics and satisfaction with e-learning courses indicated that older students, married students, and those enrolled in non-continuous bachelor’s programs reported significantly greater satisfaction with e-learning courses across all subscales, including instructor, technology, course setting, interaction, outcomes, and overall satisfaction (p < 0.05 for all). Additionally, male students and students in lower semesters reported significantly greater satisfaction with the technology and interaction subscales (p < 0.05 for all). Students studying public health, those in lower semesters, and those living in personal residences reported significantly higher satisfaction with the instructor, course setting, outcomes, and overall subscales (p < 0.05 for all). Furthermore, students who most frequently used computers or laptops in their learning, as well as students with a higher perceived adequacy of hardware resources, had significantly higher satisfaction in the outcomes subscale and course setting subscale, respectively (p < 0.05 for both).

Relationships between personality traits and satisfaction with e-learning courses

To determine the relationships between satisfaction with the e-learning course and personality traits while adjusting for the students’ demographic characteristics, the results of hierarchical multiple regression analyses are shown in Table 4. The results indicated that the personality trait of neuroticism is inversely related to satisfaction with e-learning with the instructor (p = 0.036), technology (p = 0.032), course setting (p = 0.021), interaction (p = 0.054), and outcome (p = 0.010) subscales of satisfaction, but all effect sizes were modest.

**Table 4:** Hierarchical regression analyses of students’ satisfaction with the e-learning course based on personality traits (n = 183).

Variable	Unstandard regression coefficient	Standard error	Standard beta coefficient	P-value	Model adjusted R-squared
Model 1: Satisfaction with instructor					0.28
(Constant)	4.152	0.868	---	<0.001	
Marital status (married)	0.629	0.165	0.317	<0.001	
Year of education	-0.195	0.060	-0.220	0.001	
Living status (with family)	-0.093	0.173	-0.049	0.594	
Living status (dormitory)	-0.291	0.160	-0.158	0.071	
Openness to experience	-0.112	0.125	-0.067	0.374	
Neuroticism	-0.217	0.103	-0.179	0.036	
Conscientiousness	0.084	0.150	0.050	0.576	
Agreeableness	0.169	0.145	0.098	0.246	
Extraversion	-0.155	0.113	-0.104	0.173	
Model 2: Satisfaction with technology					0.18
(Constant)	4.004	0.729	---	<0.001	
Education level (non-continuous BSc)	0.251	0.119	0.168	0.037	
Year of education	-0.123	0.054	-0.175	0.024	
Openness to experience	-0.184	0.103	-0.139	0.075	
Neuroticism	-0.185	0.085	-0.193	0.032	
Conscientiousness	-0.025	0.125	-0.019	0.843	
Agreeableness	0.266	0.120	0.194	0.028	
Extraversion	-0.048	0.094	-0.041	0.607	
Model 3: Satisfaction with course setting					0.18
(Constant)	4.276	0.789	---	<0.001	
Education level (non-continuous BSc)	0.379	0.124	0.234	0.003	
Perceived access to hardware facilities	0.289	0.084	0.257	0.001	
Major (Public Health)	0.279	0.126	0.183	0.028	
Major (Occupational Health)	0.088	0.158	0.045	0.577	
Openness to experience	-0.110	0.111	-0.077	0.325	
Neuroticism	-0.215	0.092	-0.207	0.021	
Conscientiousness	-0.221	0.140	-0.153	0.117	
Agreeableness	0.111	0.129	0.075	0.391	
Extraversion	-0.169	0.102	-0.133	0.099	
Model 4: Satisfaction with interaction					0.06
(Constant)	4.078	0.896	---	<0.001	
Education Level (non-continuous BSc)	0.390	0.138	0.225	0.005	
Openness to experience	-0.088	0.126	-0.057	0.489	
Neuroticism	-0.204	0.105	-0.184	0.054	
Conscientiousness	-0.049	0.155	-0.032	0.751	
Agreeableness	0.054	0.147	0.034	0.714	
Extraversion	-0.048	0.116	-0.035	0.681	
Model 5: Satisfaction with outcomes					0.19
(Constant)	3.243	1.019	---	0.002	
Age	0.030	0.009	0.270	0.001	
Most often used media in learning (Computer/Notebook/Laptop)	0.349	0.145	0.179	0.018	
Year of education	-0.127	0.072	-0.131	0.081	
Openness to experience	-0.022	0.147	-0.012	0.881	
Neuroticism	-0.308	0.118	-0.233	0.010	
Conscientiousness	0.181	0.177	0.098	0.310	
Agreeableness	-0.036	0.167	-0.019	0.830	
Extraversion	-0.102	0.131	-0.063	0.437	
Model 6: Overall satisfaction with e-learning					0.13
(Constant)	4.176	1.142	---	<0.001	
Age	0.024	0.010	0.200	0.021	
Year of education	-0.183	0.082	-0.171	0.028	
Major (Public Health)	0.361	0.180	0.169	0.047	
Major (Occupational Health)	0.014	0.233	0.005	0.953	
Openness to experience	-0.108	0.170	-0.054	0.525	
Neuroticism	-0.253	0.136	-0.173	0.065	
Conscientiousness	0.126	0.201	0.062	0.531	
Agreeableness	-0.084	0.189	-0.040	0.657	
Extraversion	-0.238	0.150	-0.133	0.115	

A higher score in neuroticism was associated with lower satisfaction with the e-learning course in these subscales. Additionally, beyond the trait of neuroticism, the trait of agreeableness had a significant positive relationship with satisfaction with technology (p = 0.028). Students who exhibited greater agreeableness reported greater satisfaction with technology. None of the personality traits had a significant relationship with overall satisfaction with the e-learning course.

The final regression models, adjusted for demographic characteristics, explained 6% to 28% of the variance in satisfaction scores across different subscales (Table 4). However, in these hierarchical multiple regression analyses, the personality traits explained only an additional 5% of the variability in satisfaction with the instructor, 8% in satisfaction with technology, 4% in satisfaction with the course setting, 2% in satisfaction with interaction, 5% in satisfaction with outcomes, and 2% in satisfaction with the overall subscale, all of which indicated that the relationships were of small effect size.

## Discussion

The present study aimed to investigate the relationships between the personality traits of health faculty students and their perceived satisfaction with e-learning courses. The results showed that the personality trait of neuroticism has a significant inverse relationship with satisfaction in the subscales of instructor, technology, course setting, interaction, and outcomes of e-learning courses. Additionally, greater agreeableness was associated with greater satisfaction in the area of technology. Furthermore, none of the personality traits had a significant relationship with the subscale of overall satisfaction with the e-learning course.

These results align with previous findings regarding the characteristics of neuroticism. Ahmadi et al. [18], in their study predicting the academic motivation of learners based on personality traits in paramedical students, reported that neuroticism is a predictor of lower academic motivation. Similarly, Bhagat et al. [2], in a study conducted in Taiwan investigating the effects of personality traits on learners’ perceptions of online learning, reported that the personality trait of neuroticism has a significant negative effect on learners’ participation in online courses.

Consistent with the results of the present study, Mustafa et al. [13] reported that neuroticism and extraversion were negatively related to satisfaction and the intention to adopt online learning methods, whereas conscientiousness, agreeableness, and openness to experience were positively related to satisfaction and the intention to adopt online learning methods. These authors noted that neuroticism is associated with negative emotions and that the isolation experienced during COVID-19 may have adversely affected the mental health of neurotic learners, leading to dissatisfaction with long-term online learning without any physical interaction or face-to-face learning.

In contrast to the results of the present study, Tovmasyan et al. [21] reported that neuroticism was associated with greater satisfaction, while extraversion predicted lower satisfaction with blended learning during the COVID-19 pandemic. They also found that conscientiousness, agreeableness, and openness to experience did not have a significant relationship with satisfaction.

The discrepancies observed between the present study and those of both Mustafa et al. [13] and Tovmasyan et al. [21] may be attributed to cultural and social differences in the studied populations, variations in e-learning courses delivery methods or supportive systems, demographic or contextual factors, or differences in how satisfaction has been conceptualized and measured across studies.

Satisfaction with learning often arises from a sense of achievement, enjoyment, and the relevance of the learning material. Certain personality traits can affect learning and, consequently, learner satisfaction. For example, conscientiousness is associated with work discipline, fostering interest in the subject being studied, increasing attention, and leading to the perception that studying is relatively easy. Learners with conscientious traits adopt a planned approach. They set clear goals in advance, excel in organizing their work, manage their time effectively, and hold a strong belief in the value of hard work in their studies [22]. This structured approach can contribute to satisfaction by providing a sense of achievement.

Additionally, the openness to experience characteristic is linked to critical thinking, reasoning, and problem analysis. Students with this trait are self-motivated, focused on their own growth, and seek personal understanding independent of the curriculum, making them more inclined to participate in online courses [2]. Engagement in diverse learning experiences can enhance satisfaction.

Learners with extraverted characteristics engage in human interactions and social activities [23]. These individuals possess strong social needs that motivate them to complete their courses and seek help from others when facing academic challenges [24]. These social interactions can enhance their learning experience and contribute to their overall satisfaction. Individuals with agreeableness tend to be patient and optimistic, viewing others as honest and trustworthy [23]. They also exhibit a positive attitude toward new technologies [25], which can lead to a more enjoyable learning experience.

In contrast, neuroticism is associated with attention deficits, emotional instability, self-doubt, anxiety about failure, and constant stress during study. Students with neuroticism traits often adopt a superficial learning style focused on memorization rather than comprehension, aiming merely to pass exams [22]. This limited engagement with the material can reduce their overall satisfaction. Furthermore, individuals with neuroticism may tend to be lonely and utilize social interaction websites to fulfill their social needs [26]. They often hesitate to ask questions and interact with instructors or evaluators, and they do not actively seek new experiences [25]. However, their participation in online activities may increase if they are assured of their unique contributions to the community or if evaluation is not involved [27].

In designing an effective electronic course, the characteristics of flexibility, interaction, support, and motivation should be considered [2]. To increase performance and the quality of learning, different personality traits of learners should also be taken into account by the instructor.

In the present study, 25 students (14%; 95% CI = 12%-16%) were classified as having a high level of neuroticism. Mustafa et al. [13] reported that meeting the emotional needs of students in online learning can promote positive emotions and increase their willingness to participate in an online course. Therefore, to increase the participation of learners with high neuroticism, the instructor can implement various strategies, such as providing immediate and positive feedback or clearly explaining any doubts or concerns the learners may have. This approach can help build trust in the course and enhance feelings of security and support among students [2].

Additionally, in the present study, students with higher levels of neuroticism expressed fewer competencies in software skills. Computer-related problems [20,28] and the inability to understand online media [29] have been cited as two main reasons for dropping out of online courses among adults. Studies indicate that if users are satisfied with a technology, they intend to adopt it [13]. Therefore, considering supportive strategies and training related to technology can be helpful and lead to greater satisfaction, especially among students with greater neuroticism.

According to the data analysis of the present study, none of the personality traits had a significant relationship with the subscale of overall satisfaction with the e-learning course. In contrast, characteristics such as students’ age and academic major demonstrated significant relationships with overall satisfaction. Overall satisfaction included items such as the willingness to recommend the course, future enrollment intentions, and overall contentment with the e-learning experience. These findings suggest that personality traits may not play a pivotal role in overall learner satisfaction. Instead, as reported in previous research [15,16], demographic factors such as age, education level, and marital status appear to exert a stronger influence and, in some cases, may even overshadow the effect of personality traits. This highlights the importance of considering a holistic view of learner profiles when evaluating satisfaction in e-learning contexts. Therefore, depending on the aspects of learning, e-learning courses may be more effective when designed to accommodate diverse demographic backgrounds by prioritizing factors such as major and age, rather than focusing solely on personality traits.

Study strengths and limitations

This study presents two primary strengths. First, it employed hierarchical regression models, allowing for the control of demographic confounders and providing more accurate estimates of the unique contribution of personality traits to learner satisfaction. Second, it was conducted within a health sciences educational setting, a population that is often underrepresented in e-learning research despite its critical relevance to healthcare training. Nonetheless, these strengths should be interpreted in light of several limitations. These include the small sample size for certain personality traits; the lack of detailed records on the characteristics of the e-learning courses taken by participants; the possibility of recall bias; the inherent limitations of self-report questionnaires; the study’s response rate (54%), which may introduce non-response bias; cross-cultural limitations of the assessment tools; the unique circumstances of the COVID-19 pandemic; and the constraints of a cross-sectional design in establishing causal relationships. Additionally, the specific demographic composition of the study participants, students from a health sciences faculty, with approximately 75% being female, should be taken into account when considering the generalizability of the findings.

## Conclusions

Based on this study’s findings, despite the small effect sizes, personality traits appear to be modestly associated with learner satisfaction in e-learning environments. These findings underscore the importance of considering individual personality differences when designing and delivering e-learning courses, particularly to foster a more supportive educational environment for health faculty students. Educational policymakers, planners, and academic advisors are encouraged to explore targeted strategies for personalizing digital learning experiences in ways that accommodate diverse learner profiles. While these findings offer useful insights, they should be interpreted in light of the study’s limitations. Future research with larger and more diverse samples is recommended to improve the generalizability of these findings. Additionally, assessing learner satisfaction in relation to broader learning outcomes, such as practical skills and critical thinking, along with diverse personality assessment tools, can provide a deeper understanding of how individual differences influence educational experiences.
